# Temporally resolved SMLM (with large PAR shift) enabled visualization of dynamic HA cluster formation and migration in a live cell

**DOI:** 10.1038/s41598-023-39096-4

**Published:** 2023-08-02

**Authors:** Jigmi Basumatary, Neptune Baro, Fancesca Cella Zanacchi, Partha Pratim Mondal

**Affiliations:** 1 Instrumentation and Applied Physics, Indian Institute of Science, Bangalore, India; 2 Department of Physics, University of Pisa, Pisa, Italy

**Keywords:** Diseases, Medical research, Engineering

## Abstract

The blinking properties of a single molecule are critical for single-molecule localization microscopy (SMLM). Typically, SMLM techniques involve recording several frames of diffraction-limited bright spots of single-molecules with a detector exposure time close to the blinking period. This sets a limit on the temporal resolution of SMLM to a few tens of milliseconds. Realizing that a substantial fraction of single molecules emit photons for time scales much shorter than the average blinking period, we propose accelerating data collection to capture these fast emitters. Here, we put forward a short exposure-based SMLM (*shortSMLM*) method powered by *sCMOS* detector for understanding dynamical events (both at single molecule and ensemble level). The technique is demonstrated on an Influenza-A disease model, where NIH3T3 cells (both fixed and live cells) were transfected by Dendra2-HA plasmid DNA. Analysis shows a 2.76-fold improvement in the temporal resolution that comes with a sacrifice in spatial resolution, and a particle resolution shift PAR-shift (in terms of localization precision) of $\sim$ 11.82  nm compared to standard SMLM. We visualized dynamic HA cluster formation in transfected cells post 24 h of DNA transfection. It is noted that a reduction in spatial resolution does not substantially alter cluster characteristics (cluster density, $\#$ molecules/cluster, cluster spread, etc.) and, indeed, preserves critical features. Moreover, the time-lapse imaging reveals the dynamic formation and migration of Hemagglutinin (HA) clusters in a live cell. This suggests that $short-SMLM$ using a synchronized high QE *sCMOS* detector (operated at short exposure times) is excellent for studying temporal dynamics in cellular system.

## Introduction

Standard single-molecule localization microscopy (SMLM) techniques (fPALM^1^, STORM^2^, PALM^3^, IML-SPIM^4^, MINFLUX^5,6^, dSTORM^7^, SOFI^8^, ROSE^9^, SMILE^10,11^, POSSIBLE^12^, and variants^13–17^ ) typically acquire several thousand ($> 10^{3}$ ) images of single-molecules to reconstruct a single super-resolved image. This accounts for a considerable acquisition time than typically camera exposure and readout times. In order to accelerate the overall temporal resolution, data acquisition needs to be accelerated. However, there is a limit on temporal resolution due to the fluorescence cycle time of a molecule^18^. In recent times, there have been few studies using fast blinking molecules, high excitation intensity, dark states and sCMOS camera^19–23^. In other studies, reducing acquisition time using computational techniques has shown promise. Other techniques include using fast computing engines (FPGA-GPU and CUDA) coupled with artificial intelligence have shown great potential in SMLM^24–27^. Although these techniques are helpful, they are prone to photobleaching. Here, we discuss rapid data acquisition powered by advanced sCMOS technology for capturing fast-blinking fluorescent proteins (Dendra2). The blinking behaviour of Dendra2 along with ON/OFF times is studied in detail by Avilov et al^28^. It is expected that rapid-acquisition techniques along with fast computing engines are a formidable combination and are more likely to enable real-time super-resolution imaging.

The last few years have seen a substantial advance in this direction, and many research groups have addressed this issue. A distinct approach is to increase the number of active single molecules per frame without changing the intensity, and other experimental parameters^21,29^. However, this technique comes with a cost where large activation could result in overlapping of diffraction-limited signatures of single molecules, resulting in indistinguishably of individual molecules and subsequently increasing the complexity^30^. Recent work by Lin et al. quantitatively compared the image quality for microtubules immunolabeled using Alexa Fluor 647- dye^19^. Another study by Diekmann et al. showed that two critical factors (i.e., localization precision and labeling efficiency) determine SMLM image quality. They systematically investigated the consequence of speed on image quality for different dyes (AF647, CF680, CF660C, Dy634) at different labeling efficiencies and intensities^21^. To our knowledge, photoswitchable proteins such as Dendra 2 have never been demonstrated for fast imaging and time-lapse localization microscopy and hence represent an important class of probes used in super-resolution microscopy. Specifically, photoswitchable proteins allow cloning / conjugation with protein-of-interest, which makes it suitable for studying biological processes (such as disease progression^31–33^) and helps in understanding the underlying mechanism.

A recent technique based on deep learning suggests accelerating the post-acquisition time without compromising spatial resolution in 2D, or 3D localization of each blinking molecule per frame^34,35^. Another technique that promises to improve temporal resolution is an acquisition device based on sCMOS technology that excels EMCCD technology by reducing the read-out time without compromising on the quantum efficiency^21,27^. A recent dSTORM imaging of fixed cells using sCMOS camera with Alexa 647 dye as a probe has shown potential for fast SMLM^36^. Moreover, STORM imaging is also performed at low-to-high speeds for optimal resolution and image quality^21,37^.

In general, probing complex biological processes necessitates rapid data acquisition. This has many benefits, including observing millisecond / sub-millisecond dynamics and substantially reducing long acquisition times. These factors have consequences on signal-to-noise ratio, image background, and system drift. Here, we report a simple method, implemented by reducing the data acquisition time on conventional single molecule detection architectures, to overcome the bottlenecks that plague many biophysical processes occurring at faster time scales. Specifically, we study HA clusters and related dynamical changes over time in a cellular environment. To realize this, a short exposure ($\sim 5~{\text {ms}}$) acquisition method (powered by sCMOS detector) is proposed. The goal is to understand and evaluate the impact of the short exposure time on the localization precision and its consequence on clustering analysis. Overall, the method improves temporal resolution at the cost of localization precision while preserving biophysical parameters related to cluster formation (e.g., cluster density, $\#$ molecules / cluster, cluster spread, etc).

**Figure 1:**
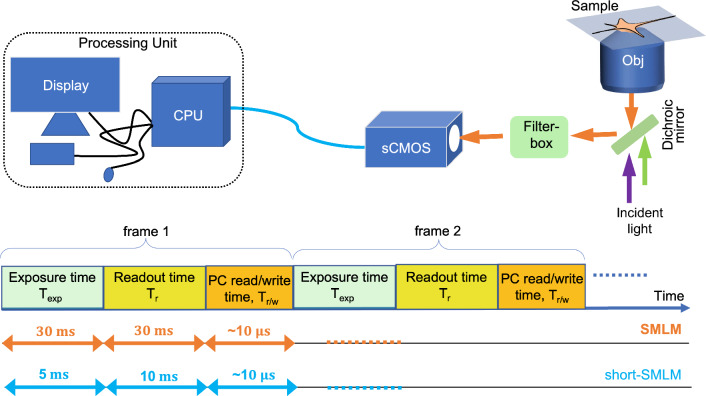
Key hardware system involved in SMLM data acquisition process. Time taken by a single frame acquisition is the sum of time taken by each sub-system in the data acquisition process. Here, “Read/write” is abbreviated as *r*/*w*.

**Figure 2:**
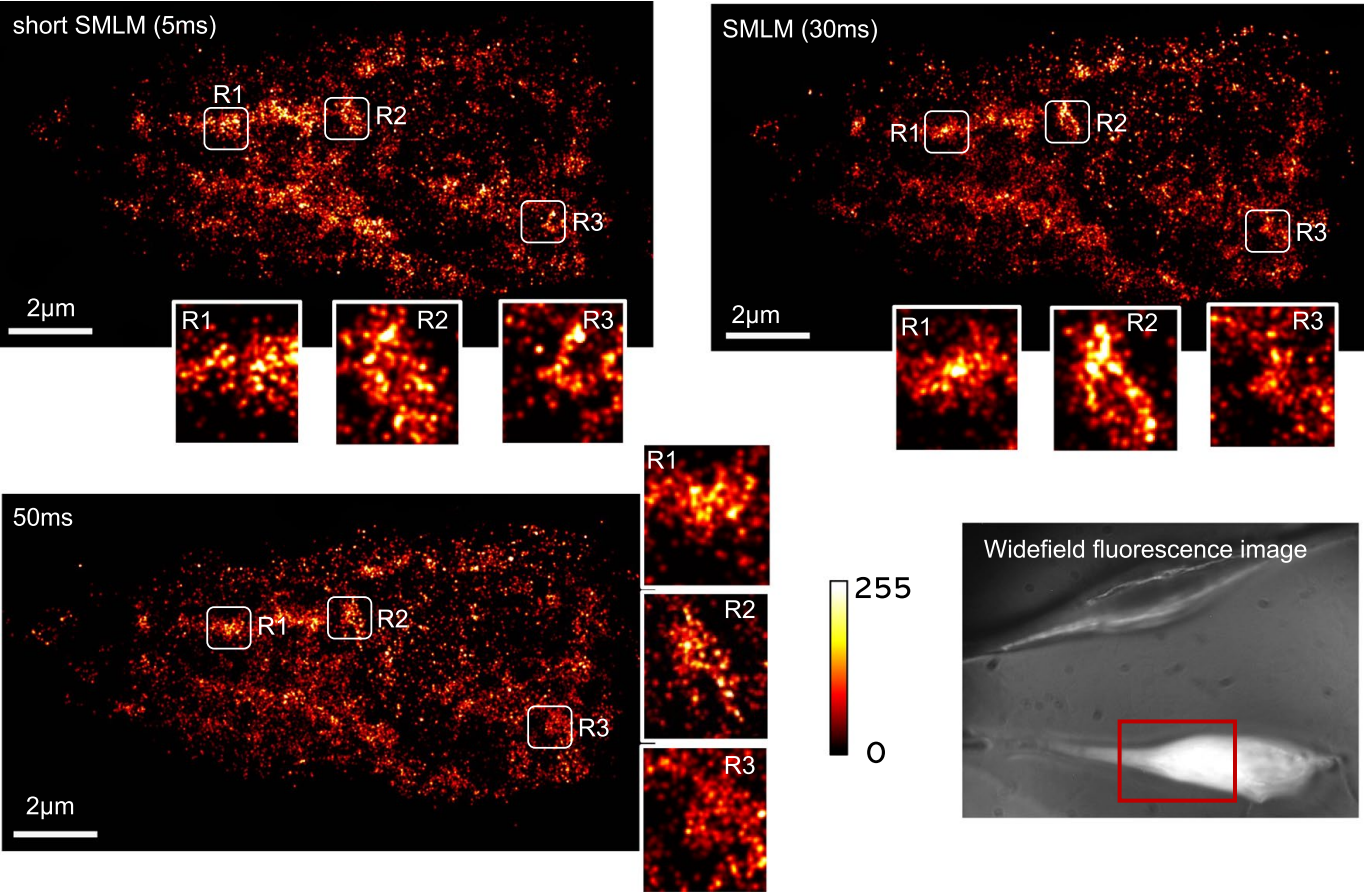
Super-resolved image of HA molecules 24 h post transient D2HA transfected NIH3T3 cells for 5 ms, 30 ms and 50 ms exposed acquired data using Zyla 4.2 plus (Andor). $2\times 2$ binning is used while acquiring data. Each reconstructed image contains approximately, 20, 000 HA molecules. Corresponding transmission and fluorescence (at, 510 nm) images are also shown.

**Figure 3:**
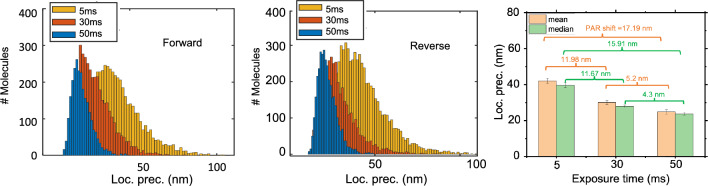
The localization precision plots of the reconstructed images obtained for both forward ($5~{\text {ms}} - 30~{\text {ms}} - 50~{\text {ms}}$) and reverse ($50~{\text {ms}} - 30~{\text {ms}} - 5~{\text {ms}}$) schemes. The corresponding bar-plots for localization precision for forward and reverse cases along with the Particle Resolution shift (PAR-Shift) are also shown.

**Figure 4:**
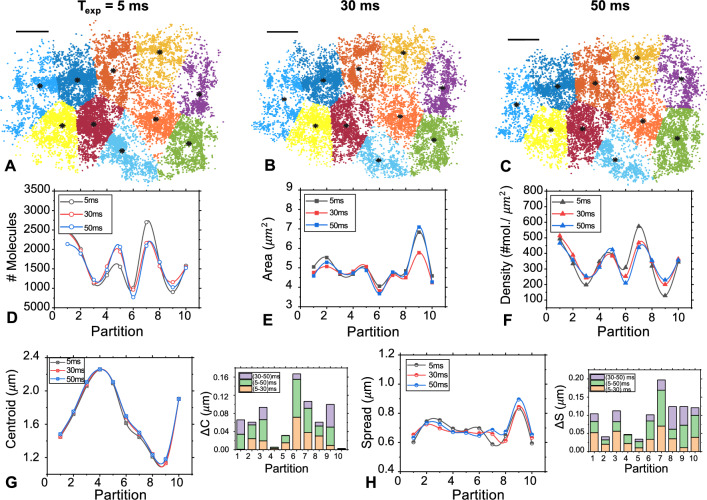
(**A**–**C**) Partioned (using K-medoid method) super-resolved images of HA molecules distributed in the cell for recordings at $5~{\text {ms}}$, $30~{\text {ms}}$ and $50~{\text {ms}}$. (**D**–**F**) The corresponding parameters such as, $\#~molecules$, area and density per partition are also estimated. (**G**, **H**) The centroids and spread of partitions along with pair-wise ($50 \& 5$, $30 \& 5$ and $30 \& 50$) change of these parameters are also shown using bar-plots.

**Figure 5:**
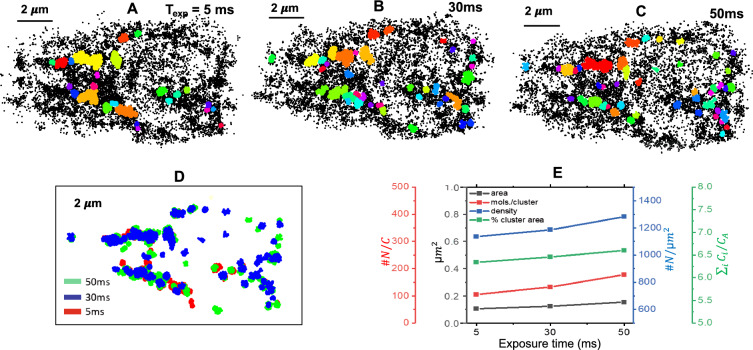
(**A**–**C**) DBSCAN estimated molecular clusters for data obtained at short exposure time along with standard SMLM and large exposure time ($50~{\text {ms}}$). (**D**) An overlay of the clustered localizations, color-coded by the integration time. (**E**)The plots show average value of estimated biophysical parameters (number of HA molecules per μm^2^, number of molecules per cluster ($\# N / C$), cluster area (μm^2^) and clustered fraction $\sum _i C_i / C_A$ ) relevant to formed molecular clusters, where $C_i$ and $C_A$ correspond to total cluster area and cell area, respectively.

**Figure 6:**
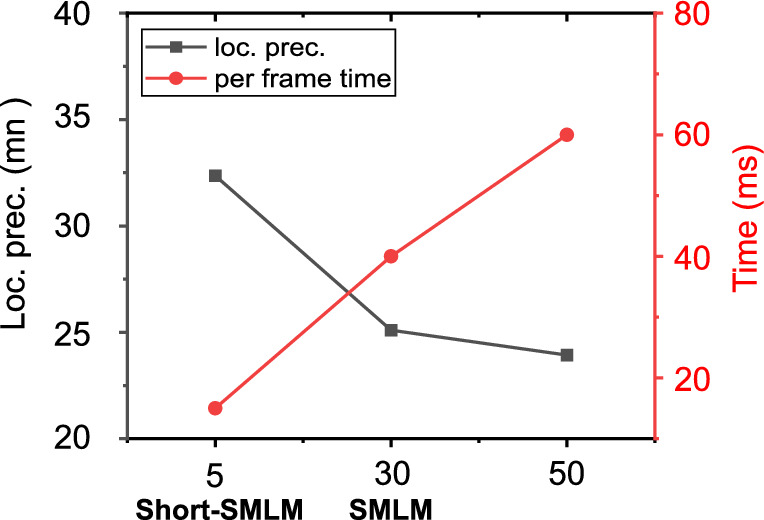
Time per frame and localization precision plots. It is evident that gain in temporal resolution results in poor spatial resolution and vice-versa (see, black curve). The red curve (actual integration time) correspond to increase in per frame time with exposure time as per the scheme in Fig. 1.

**Figure 7:**
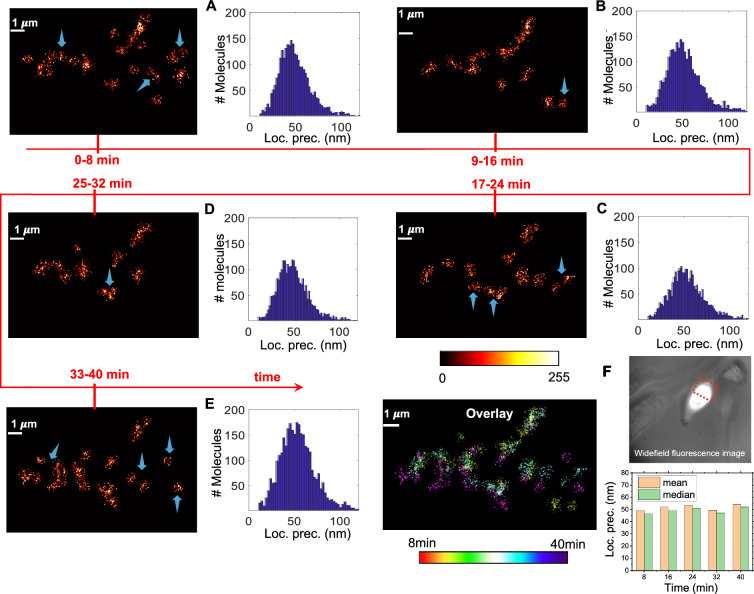
Live cell imaging of Dendra2-HA transfected cells. Unclustered molecules are removed from the images for further analysis. Alongside localization precision is also shown. Data is divided into 5 parts (**A**–**E**) based on time acquisition (0–8 min, 9–16 min, 17–24 min, 25–32 min, 33–40 min). Alongside, overlay of all the image (by time points) is also shown. (**F**) Localization precision versus time is also shown along with the fluorescence image of transfected cell.

## Results

The proposed technique is employed to improve the temporal resolution of standard SMLM by reducing the exposure time of the detection device (sCMOS camera). This is based on the assumption that a substantial fraction of molecules blinks for less than the average blinking period ($30~{\text {ms}}$)^38–40^. Our approach is to capture these fast-blinking molecules with a lifetime of less than $\le 5~{\text {ms}}$^1^. In the present technique, the sCMOS camera is operated at short exposure times for fast frame rates. This helps in capturing fast-blinking molecules. A schematic diagram giving acquisition details for $short-SMLM$ system and its comparison with standard SMLM is shown in Fig. 1.

Figure 2 displays the reconstructed super-resolved images of Dendra2-HA transfected NIH3T3 cell (see, transmission image). The transfection protocol and cell culture conditions are detailed in methods section. To characterize the impact of detector exposure time on single molecule signatures, we collected images with increasing order, $5~{\text {ms}},~30~{\text {ms}}, 50~{\text {ms}}$ as shown in Fig. 2. A total of 20,000 molecules are detected from approximately 5000 frames. The corresponding localization plots are shown in Fig. 3. The plots indicate a reduction in localization precision with decrease in exposure time, which is due to less number of detected photons per molecule. Specifically, we note a PAR-shift of $11.86~{\text {nm}}$ as compared to standard SMLM. When the same experiment is repeated in decreasing order, the trend continues, although the average localization have changes a bit, and a PAR-shift of $10.19~{\text {nm}}$ is noted. This suggests that, a sacrifice in localization precision leads to improvement in the temporal resolution. The exposure time has a consequence on the localization precision. This suggests a PAR-shift towards diffraction limit when compared to standard SMLM and beyond (at $50~{\text {ms}}$). Overall, an average PAR-shift (in terms of localization precision) of $11.86~{\text {ms}}$ is noted throughout the experiments.

To access the resolution of reconstructed images, we have carried out Fourier Ring Correlation (FRC) analysis^41,42^. FRC is preferred over other metric since it does not require any prior information, and the metric can be computed in real-time. FRC is based on the fact that every system has an effective cut-off frequency, and resolution is determined by high spatial frequencies supported by the system. Fig. S4-1 shows the FRC analysis for 5 ms, 30 ms and 50 ms for which the corresponding FIRE values are 60.36 nm, 93.99 nm, and 98.43 nm, respectively. A standard threshold of $1/7=0.143$ is chosen The spatial frequency where the FRC falls below the threshold is defined as cut-off frequency and the resolution is calculated. A detailed analysis for both the cases can be found in Supplementary 4.

To access the impact of short exposure time on the single molecule clusters, we employed our technique to visualize partitions in Dendra2-HA transfected NIH3T3 cells, and experimental conditions mostly used for SMLM. The results are shown in Fig. 4 for the froward scheme i.e, beginning from short ($5~{\text {ms}}$) to long exposure ($50~{\text {ms}}$). The complete data acquisition for both forward and reverse measurement schemes are detailed in Supplementary 1. The schemes are employed to ensure bias-free recording and uniformity among the datasets at varying exposure times. For ensuring similarity, we have used K-mediod based unsupervised segmentation^43^, and related parameters (centroid, spread and size (area) of the partition) are estimated. From the single molecule maps (with partitions labelled by different colors and their centroids), consistency can be ascertained visually (see, Fig. 4A–C). In addition, corresponding parameters related to the number of molecules, area and density of partitions are also estimated (see, Fig. 4D–F). These parameters suggests bias-free data acquisition at varying exposure time for both forward and reverse cases, even though experiments are performed at different time-points. Figure 4G shows the location of centroids for the partitions. For a majority of these partitions, the shift in centroid location is less than 18 *nm*, and the shift in mean spread is $58~{\text {nm}}$ (see, Fig. 4H). This still gives an excellent value considering the fact that molecules are detected and localized independently for sequential data collection at different exposure times ($5~{\text {ms}}, ~30~{\text {ms}}, ~50~{\text {ms}}$). Moreover, relative small changes in centroid $\Delta C$ and spread $\Delta S$ (pairwise, between 5 and 50 ms; 5 and 30 ms; 30 and 50 ms) indicate similarity of partitions (see, Fig. 4G,H). Overall, Fig. 4 suggests bias-free data collection for experiments performed at different time-points and at varying exposure times.

The immediate next step is to understand clustering process in transfected NIH3T3 cells to understand HA dynamics in Influenza-A model. We employed DBSCAN clustering technique for the single molecule data recorded at $5~{\text {ms}}$ and compared the same with standard SMLM ($30~{\text {ms}}$). DBSCAN has the distinct ability of accounting for only the clustered data and overlooking unclustered data. Here we have chosen a cut-off for HA-HA distance of $80~{\text {nm}}$ and a minimum of 35 HA molecules for recognizing clusters. The corresponding HA-clusters are shown in Fig. 5A–C Although the proposed study focus on short exposure ($5~{\text {ms}}$) data acquisition, we have also acquired data at relatively longer exposure ( $\sim 50~{\text {ms}}$) for consistency. To ensure that same clusters are observed for all the exposure times, an overlay of the clustered localizations are shown in Fig. 5D, where integration time is color-coded. In addition, biophysical parameters such as, cluster density (1138 mol./μm^2^), cluster area (0.105 μm^2^), number of molecules per cluster (105), and clustered fraction or relative cluster fill ratio ($6.33 \%$ per cell) are also determined. The parameter values are in consistent with the study reported in literature^32^. We did not see substantial change in these parameters for short exposure times especially when compared to standard SMLM. This is evident from the plots shown in Fig. 5E. Visually, the cluster appears consistent at all exposure time. This confirms the fact that structural information related to HA-clusters is preserved at short exposure times.

To understand the repercussions of short exposure time on image quality, we calculated the mean localization precision. It is quite evident that, localization precision suffers at short exposure time. Specifically, a decrease in exposure time as compared to standard SMLM, results in a slight decrease in localization precision (or spatial resolution) of about $11.86~{\text {nm}}$ (corresponding to a PAR-shift towards classical diffraction limit, $\sim \lambda / 2$). So, the cost of fast super-resolution imaging is a partial sacrifice of spatial resolution (Fig. 6). However, this did not significantly altered our analysis on single molecule clustering for the proposed disease model (Influenza-A model). A detailed study on another sample is discussed in Supplementary 1and 2. This indicates that, a loss in localization precision may be acceptable for analysing single molecule clusters and its dynamics in order to fetch gain in temporal resolution. This also goes on to suggest that, there is some sort of uncertainty relation between localization precision and temporal resolution as far as single molecule imaging is concerned.

To substantiate the advantage of $short-SMLM$ for high temporal resolution, experiments are carried out on live NIH3T3 cells. Post transfection with Dendra2-HA plasmid, the cells were imaged for a long time (about 40 min) and the data is divided into 5 sets, each of 8 min duration. Approximately, 5000 images are collected for each set and 3000 single molecules are identified. Subsequently, super-resolved images are reconstructed for each set and localization precision is plotted as shown in Fig. 7. Cluster analysis is carried out using DBSCAN algorithm and non-clustered molecules are removed. Visual analysis and overlay of all the images (by time points) evidences temporal changes in HA-clusters that form post transfection, as indicated by blue arrows in Fig. 7. This suggests for the first time, the dynamical nature of clustering process where HA-clusters form and/or migrate during early stages of viral infection in a cellular system (Influenza-A model). The localization precision versus time plot (see, Fig. 7F) does not show significant change in the average localization precision indicating the potential of $short-SMLM$ for prolonged super-resolution imaging and studying rapidly occurring events (here, collective dynamics of HA molecules) in a live cell.

## Discussion

We demonstrated $short-SMLM$ technique for accelerating single molecule based super-resolution imaging. With the advancement in sCMOS camera technology, it has become possible to collect data at shorter exposure times; of-course this cannot be less than the temporal resolution limit in fluorescence microscopy^18^. The fact that single molecule blinking occurs at varying time scales ranging from a few to tens of milliseconds, the data acquisition process can be accelerated by collecting images at a few milliseconds as compared to standard SMLM^1^. This necessitates the detection of fast-blinking molecules. The technique is beneficial for visualizing biophysical processes (clustering, migration, binding, and diffusion at single-molecule level). In general, data acquisition at large exposure time (used in standard SMLM) wipes out events (blinking and fluctuations) that occur at relatively short exposure times. Moreover, relatively long exposure frequently used in SMLM may significantly misrepresent locations of single molecule clusters that change dynamically. A detailed study related to the blinking characteristics of Dendra2 with exposure time is incorporated in Supplementary 3.

Here, we report recording at short data acquisition times, which is 2.76 times less than standard SMLM. Results show that there is a shift in localization precision (resolution) towards the classical diffraction limit. Specifically, a PAR-shift (in terms of localization precision) of $11.86~{\text {nm}}$ is noted as compared to SMLM. However, the shift is tolerable when accessing the benefits of high temporal resolution. Further analysis of single molecule distribution has revealed a high degree of consistency in data collection at a short exposure time. This is further ascertained by the estimated parameters such as the number of molecules, the area, and the density of single molecule clusters. The fact that centroid and distribution spread is negligible talks a lot about the consistency and reliability of data collection at short exposure times.

To understand cluster formation in a live cell transfected with Dendra2-HA plasmid DNA(Influenza-A disease model), DBSCAN clustering technique is employed. Results show consistency in cluster formation for data collected at an exposure time of 5 *ms* to as large as 50 ms. In addition, DBSCAN has enabled the determination of critical biophysical parameters such as cluster density, cluster area, number of molecules per cluster, and the clustered fraction in a cell. The time-lapse imaging is performed on a live transfected cell to visualize the temporal dynamics of HA clusters (see, Fig. 7). Results indicate the formation and migration of HA clusters with time. These parameters are directly associated with the infectivity rate and disease progression in a disease model (here, Influenza-A)^32,44^. So, $short-SMLM$ facilitates the study of the temporal dynamics of viral particles (here, HA single molecules), which is a step closer to early diagnosis and therapy. At a single molecular level, this means using potential drugs for disrupting HA-clusters during Influenza-A infection. Moreover, we noted a relation between temporal resolution and localization precision, i.e., an increase in temporal resolution (short exposure time) resulted in a proportional decrease in localization precision and vice-versa. Considering the benefit of temporal resolution, the PAR-shift (towards diffraction-limit) may be acceptable for gaining insight into the rapidly occurring events in a cellular system.

## Materials & methods

### Imaging system and low-exposure data acquisition

A basic cartoon of data acquisition processes involved in a typical SMLM microscopy system is shown in Fig. 1. The time involved for specific processes is also represented, i.e, the exposure time $t_{exp}$, the read-out time $t_r$, and the PC read-write time ($t_{r/w}$). Specifically, the frame transfer rate (FR) for *sCMOS* technology can be expressed as,

1 $$\begin{aligned} FR=\frac{1}{t_{exp} + t_{r} + t_{r/w} } \end{aligned}$$

where, read-out time is given by $t_{r/w}=\frac{1}{ N + Dr}$, with *N* as the number of pixels ($\#$ pixels) and $D_{r}$ is digitizer rate. Note that, frame-rate can be increased by reducing exposure-time and/or frame-size. Practically, this translates to identifying fast-blinking molecules (molecules with short-lifetimes) that has the potential to accelerate reconstruction of super-resolved image.

A typical SMLM is customized as required by the low exposure data acquisition. Two lasers, activation laser 405 nm(Oxxius, France) and readout laser 561 nm(Oxxius, France) laser, are combined with dichroic mirror. An optotune lens(EL-10-30-CI-VIS-LD-MV,Edmund optics) focuses co-linear combined beams into the back aperture of an oil immersion 1.3NA 100$\times$(Olympus) objective lens of the inverted IX81 Olympus microscope. By changing the focal length of the optotune lens, the field of view can be varied^45^. A dichroic filter attached to a filter cube distinguishes the emitted fluorescence signal from the illumination beam. The fluorescence signal is collected by the same objective and focused by the microscope tube lens. Additional magnification factor of 2.67X is introduced by a combination of two tube lenses $f_{1}$ = 75 mm and $f_{2}$ = 200 mm before focusing on sCMOS camera (Zyla 4.2 plus, pixel size 6.5 μm, Andor). The imaging system has a total magnification of 267. Notch filter set (405 nm and 561 nm Shemrock, USA) is used to cut-off unwanted signals. The intensity of activation and illumination beams can be finely controlled by a combination of polarizing and quarter wave-plates, which are installed in the path of each laser beam^11,12,46^. In addition, a separate optical arm is integarted with the system (with a blue light source (470–490 nm) ) to visualize trensfected cells. The system can switch between fluorescence and super-resolution mode as required without disturbing the sample stage.

### NIH3T3 cell culture and transfection

NIH-3T3 fibroblast cells obtained from our collaborator Prof. Upendra Nongthomba (Biological Sciences, Indian Institute of Science, Bangalore, India) were used for the experiment. We choose to work with Influenza-A model, where NIH-3T3 fibroblast cells were transiently transfected with Dendra2-HA plasmid. To begin with, the cells were thawn from preserved specimen in liquid Nitrogen ($-196 \circ C$) are cultured on growth media (90$\%$DMEM + 9$\%$ calf bovine serum + 1$\%$ penicillin-streptomycin). After 24 h post culture, a new growth median was added after discarding washed 1X phosphate-buffered saline (PBS). Seeding is done once the cell confluency is greater than 80$\%$density with cell count about $10^{5}/{\text {cm}}^{2}$. After two passage seeding, transfected cells are grown in a 35 mm disc(with coverslip). 80–90$\%$ confluent grown is maintained for transfection. We followed Lipofectamine 3000 (Life Technologies, Invitrogen) transient transfection protocol for Dendra2HA transaction in NIH3T3 cell. For live imaging, the cells were washed with 1X PBS and growth media is added after 24 h of transfection. However, for fixed cell imaging, cells were washed with 1X PBS and fixed with 3$\%$ formaldehyde, and incubated for 15 min at room temperature. Subsequently, after discarding liquids, a drop of mounting media fluorsave (Thermofisher Scientific) is used and on the top of rectangular coverslip No.0(BlueStar) place. The fixed cells were then air-tightened with nail paint for long time preservation and investigation.

### Imaging and data acquisition

We studied Dendra2 proteins conjugated with influenza viral protein Hemagglutinin (Dendra2HA) as a photoactivable protein for probing HA dynamics in a transfected cell. Upon UV-violet (360–420 nm) irradiation the protein switches to red fluorescent state. Non-activated Dendra2HA can be visualized at excitation-emission maxima at 480/507 nm, whereas activated Dendra2HA protein possesses excitation/emission maxima at 553/ 573 nm. The fluorescent protein Dendra2HA is key to understand spread of the influenza virus in mammalian cells (such as NIH3T3 cells). This has been associated with controversial cholesterol-rich lipid rafts. Influenza virus is assembled from raft domains in the plasma membrane. Expression of HA^47^, cluster size, number, and density are quantitative parameters in understanding the infection and release of buds from infected cells^48^. Exiting systems^31,49^ have shown the presence of clusters of HA molecules with high exposure time only. Upon photo-activation followed by excitation 561 nm, with desired exposure time, the fluorescence from single molecules is recorded using sCMOS. The localization precision was calculated using Thompson’s relation^50^. During experimentation, the average background noise is found to be $\sim 27$ photons / pixel, and the average photon number is $\sim 170$. The images are recorded on a $512\times 512$ pixels, and the image pixel size is calculated to be 82.15 nm (for a system magnification of 267X). In addition, sCMOS comes with a benefit of reduced data transfer-rate ($\sim 10 ~{\text {ms}}$) as compared to EMCCD that has a fixed rate of $\sim 30~{\text {ms}}$ for a detection window of $512 \times 512$ pixels. We performed acquisition at an exposure time of, 5 ms, 30 ms and 50 ms with a time gap of 1 min between consequent experiments / data recording. It may be noted that, an exposure of 5  ms essentially translated to an effective single frame (of size, $1024 \times 1024$ pixels) acquisition of $15~{\text {ms}}$ (an exposure time of $5~{\text {ms}}$ and a readout time of $10~{\text {ms}}$ ) . Experimentally the data acquisition speed(considering only exposure time, readout time, and neglecting speed of system data writing/reading) of 5 ms, 30 ms, 50 ms exposed data sets are 66 fps, 25 fps, and 15 fps, respectively. We acquired a forward data set, which is a loop of 5 ms-30 ms-50 ms-30 ms-5 ms exposure acquired data set, and reverse data set is the loop of 50 ms-30 ms-5 ms-30 ms-50 ms exposure acquired data set on the different cell with the same camera configuration (see, Supplementary 4). Overall, we acquired a total of 5 set of data for the study. A pixel binning of 2 $\times$ 2 is used throughout the data acquisition process. Pixel binning techniques combine the multiple pixels into a single pixel, thereby larger exposed area hence the collection of more photons with reducing in readout noise even though there is low exposure time. Data acquisition is made through the Andor Solis platform, which directly writes data into SSD installed in the PC system. All acquired data set is saved as a stack of 16-bit .tif format. The laser power used: 561 nm is 49 mW and 405 nm 5 μW. In the case of 5ms exposure data acquisition, the power of 405 nm and 561 nm lasers were kept at 6 μW and $20~{\text {mW}}$, respectively (see, Supplementary 5).

Single molecules are counted from the recorded data after standard post-processing that includes background subtraction from the raw data and thresholding^32,51–53^. Specifically, the background is dynamically estimated from the raw data itself, and we have chosen a threshold that is 5 times the background. The counting method considers sparse fluorescent bursts within the characteristic blinking period as one molecular count with repeated molecule counts condensed into one^32,53^.

Each 16-bit .tif frame is initially converted into photon count using the photon to pixel ratio. The photon to pixel ratio is the measure of the slope of variance vs. mean intensity^50^. Single-molecule reconstruction of the single molecules is done using einzel code on matlab2021b^1,10,11^ . The reconstruction follows the standard steps. The first step of the process is background subtraction using rolling ball algorithm^54^. Background subtraction is important because it does not contribute to determining emitter position instead adds up to signal, thereby decreasing in SNR. Following background subtraction, identification of probable molecule is made by an intensity threshold. This process is to search probable molecule which is greater than the threshold (T1) through the given frame. All the pixel’s value above T1 is tabulated. A window of between 3 $\times$ 3 pixels and 7 $\times$ 7 pixels having high intensity as the origin with at least three pixels having intensity value greater than the minimum threshold is passed after the threshold for determining coordinate and intensity within it. For each object detection, the respective centroid calculation gives the initial guess coordinates for Gaussian fitting,

2 $$\begin{aligned} G(\mu , \sigma ) = A~e^{\left[ \left( x-\mu _x\right) ^2 + \left( y-\mu _y\right) ^2\right] / \sigma ^2} \end{aligned}$$

where, the amplitude of Gaussian fit gives number of photons detected from that molecule. All the molecules are of same size and the intensity is decided by the number of photons.

## Supplementary Information

Supplementary Information 1.

Supplementary Information 2.

Supplementary Information 3.

## Data Availability

Raw data used in the study are available upon request to the corresponding author.
