# Supplementary material for: Temporally resolved SMLM (with large PAR shift) enabled visualization of dynamic HA cluster formation and migration in a live cell
Source: Sci Rep. 2023 Aug 2;13:12561. doi: 10.1038/s41598-023-39096-4 (PMC10397235; doi:10.1038/s41598-023-39096-4)
Supplement: Supplementary file 1 — Supplementary Information 1. [file 41598_2023_39096_MOESM1_ESM.pdf]

Supplementary Material: Temporally Resolved SMLM (with large PAR shift) Enables Visualization of Dynamic HA Cluster Formation and Migration in a Live Cell

Jigmi Basumatary[1], Neptune Baro[1], Francesca Cella Zanacchi[2], Partha Pratim Mondal[1]

[1]Instrumentation and Applied Physics, Indian Institute of Science, Bangalore, India

[2]University of Pisa, Pisa, Italy

Supplementary 1- 5

Supplementary 1. Sequential data collection for forward (5ms – 30ms – 50ms) and reverse (50ms – 30ms -5ms) schemes

Supplementary 2. DBSCAN clustering and Biophysical parameter estimation

Supplementary 3. Dendra2 Blinking Statistics

Supplementary 4. FRC Analysis for 5 ms, 30 ms and 50 ms.

Supplementary 5. Calibration of Exposure Time with Laser Intensity

Supplementary Videos

Supplementary Video 1. Recorded raw data 1 for cell #1 (forward & reverse schemes) at varying exposure times.

Supplementary Video 2. Recorded raw data 2 for cell #2 (forward & reverse schemes) at varying exposure times.

Supplementary 1: Sequential data collection for forward (5ms – 30ms – 50ms) and reverse (50ms – 30ms -5ms) schemes

In this supplementary, we elaborate on low exposure super-resolution imaging and compare the results with standard SMLM. Specifically two schemes are adopted, (1) low-to-high (5ms-30ms-50ms) exposure time, and (2) high-to-low (50ms-30ms-5ms) exposure time. The two schemes are employed on the same region of the cell to ensure uniformity throughout the imaging process involving various exposure times. This enables bias-free evaluation of HA clustering in Dendra2-HA transfected NIH3T3 cells. Fig. S1-1 shows the reconstructed single molecule image for a sample cell (Cell #1), first with increasing exposure time followed by decreasing exposure time in a single dataset. Overall, a total of 20,000 single molecules are recorded from approximately 5000 images and super-resolved image is reconstructed for each exposure time.

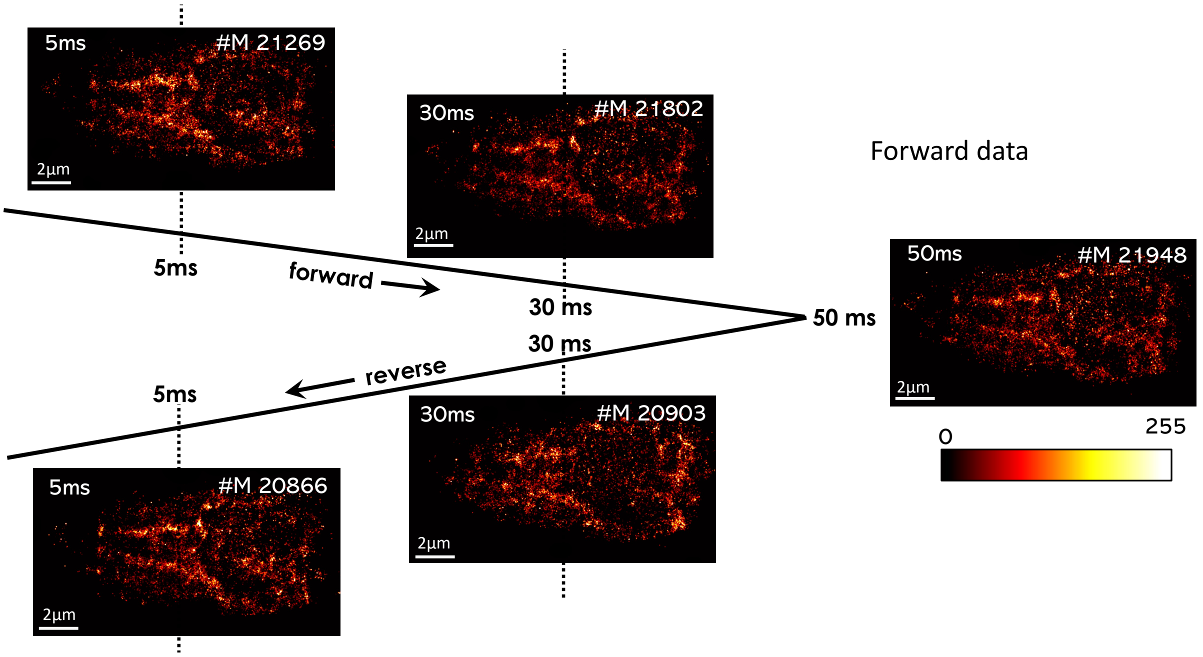

FIG. S1-1 Super resolved images of 3T3 cell transfected with D2HA plasmid for ascending order acquisition i.e, 5ms-30ms-50ms-30ms-5ms. All data sets are recorded from the same region of the cell with a time gap of 2 minutes between two consecutive data sets.

The above procedure is reported for another sample cell (Cell #2), and the reconstructed super-resolved images are shown in Fig. S1-2. Alongside, fluorescence image is also shown. It is visually evident that the distribution of single molecules is not significantly different at low exposure times as compared to data taken for standard SMLM (30 ms). Moreover, we calculated average localization precision (see, Table in Fig. S1-2), which shows similar values for both forward and backword values. This implies the significance of fast imaging for better temporal resolution without effecting localization precision of reconstructed super-resolved image.

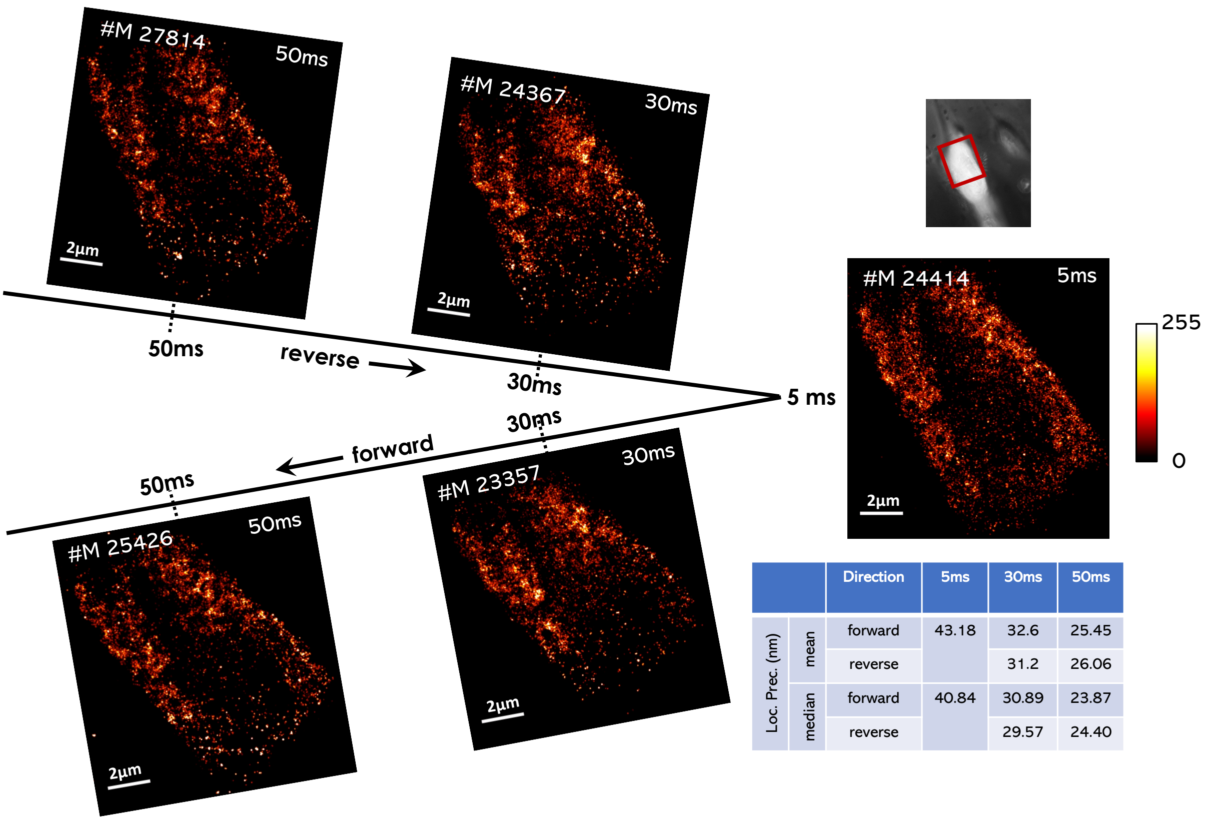

Loc. Prec. (nm)		Direction	5ms	30ms	50ms
	mean	forward	43.18	32.6	25.45
		reverse		31.2	26.06
	median	forward	40.84	30.89	23.87
		reverse		29.57	24.40

FIG. S1-2 Super-resolved images of 3T3 cell transfected with Dendra2-HA plasmid for descending order data acquisition, i.e, 50ms-30ms-5ms-30ms-50ms. All data sets are recorded at the same region of the cell with a time gap of 2 minutes between two consecutive data sets.

Supplementary 2: DBSCAN clustering and biophysical parameter estimation

It is of paramount importance to ensure that the characteristics of the underlying biophysical processes remain unaltered at low exposure times. To substantiate, we have carried out cluster analysis for Cell #2 using DBSCAN technique as shown in Fig. S2 [1-3]. DBSCAN clustering is performed with ε-value of 80 nm (chosen distance of $<$ 80 nm between two HA molecules) and a minimum of 35 molecules for identifying clusters. From the visual inspection of clustered data (see, Fig. S2[A]) and the parameter estimation (see, Fig. S2[B]), the characteristics of single molecule clusters have not significantly changed. This indicates potential of low exposure super-resolution imaging for estimating key biophysical parameters.

A.

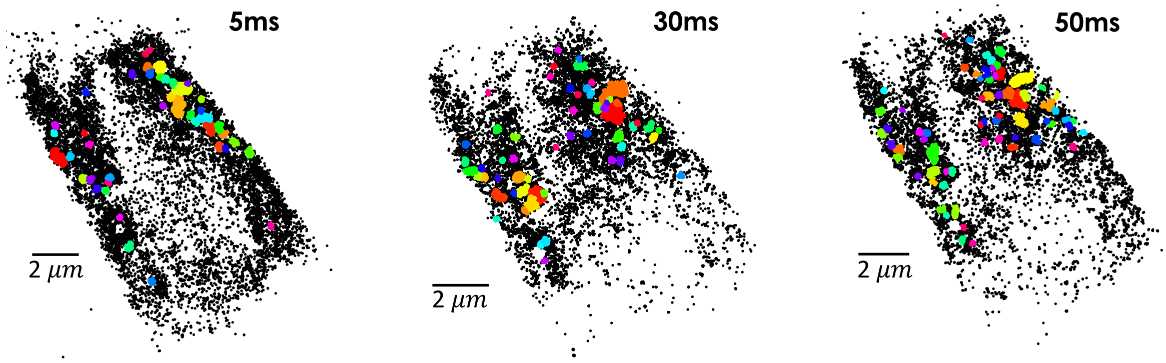

B.

Exposure (ms)	Density (#mol./μm^2)	avg. area (μm^2)	avg. mols. Per cluster	total cluster area (μm^2)	total clustered mols.	cluster area fraction (%)
5ms	1009.87	0.101	93.86	4.36	4036	6.38
30ms	1851.38	0.072	112.51	4.07	6301	5.95
50ms	1757.30	0.0664	94.83	4.38	6259	6.41

Fig. S2 [A] DBSCAN cluster of for data acquisition at varying exposure times (5 ms, 30 ms, 50 ms). Coloured points clouds are clusters constituting molecules. [B] Table shows the cluster parameters (cluster density, average cluster area and average number of molecules per cluster).

References:

1. Ester, M., H.-P. Kriegel, J. Sander, and X. Xiaowei. "A density-based algorithm for discovering clusters in large spatial databases with noise." In Proceedings of the Second International Conference on Knowledge Discovery in Databases and Data Mining, 226-231. Portland, OR: AAAI Press, 1996.

2. S S Li, "An Improved DBSCAN Algorithm Based on the Neighbor Similarity and Fast Nearest Neighbor Query", IEEE Access, pp. 99, 2020.

3. Mingrui Zhang, Use Density-Based Spatial Clustering of Applications with Noise (DBSCAN) Algorithm to Identify Galaxy Cluster Members, IOP Conf. Ser.: Earth Environ. Sci. 252, 042033 (2019).

Supplementary 3. Dendra2 Blinking Statistics

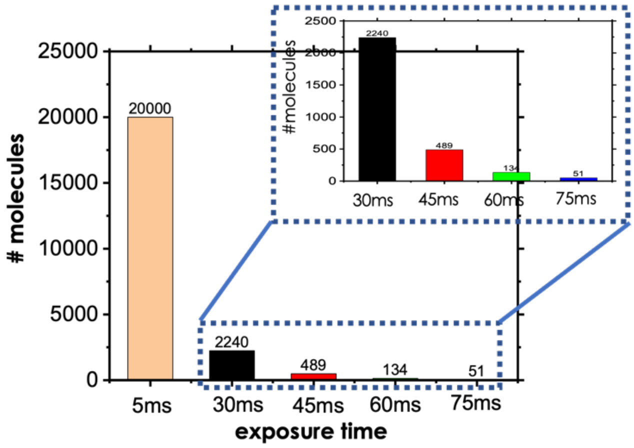

Fig. S3-1. Blinking statistics of actual number of detected Dendra2 molecules at an exposure time of 5 ms, showing more localizations per interval time.

Single molecule counts are obtained from the molecules which are spatially separated enough to be detected individually in each frame [1]. In a frame, counting of single molecule PSF is carried out by inscribing in a single square region of interest (ROI), whose width is 3 times the FWHM of the measured PSF. The counting excludes the regions of interest (ROI) that are closer than 1.7 times the half-width from any other ROI. Moreover, the repeated molecules in subsequent frames are identified by position(centroid), and the repeated molecules in subsequent frames are condensed to a single count.

Fig. S3-1 shows the single molecules statistics recorded at an exposure time of 5ms. For a given recorded data, 20000 molecules (at 5 ms) are found to be non-repeated, and a small fraction of approximately 2914 molecules (for ≥ 30 ms) are repeated in subsequent frames which are condensed while counting. Non-repeated molecules are used for reconstructing super-resolved image.

References

[1] Samuel T. Hess, Thanu P.K. Girirajan , Michael D. Mason , Ultra-High-Resolution Imaging by Fluorescence Photoactivation Localization Microscopy Author links open overlay panel, Biophysical Journal 91(11) 4258–4272.

Supplementary 4. FRC Analysis for 5 ms, 30 ms and 50 ms.

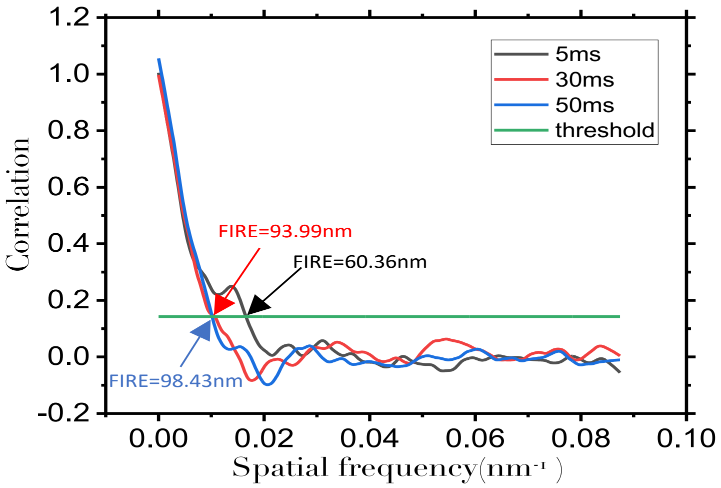

Fig. S4-1. FRC analysis for 5 ms, 30 ms and 50 ms for which the corresponding FIRE values are, 60.36 nm, 93.99 nm and 98.43 nm, respectively.

To quantify the resolution of images we used Fourier Ring Correlation (FCR) analysis. FRC is a method for calculating the correlation between two images as a function of spatial frequency. It can be viewed as a measure of the resolution of SMLM image, when applied to a pair of super-resolved images produced by separating the list of localization coordinates into two subsamples. The two SMLM subimages are correlated by multiplying their Fourier transformations (F_x & F_y), and the FRC_{xy} is calculated by summing over concentric rings r_i followed by normalizing the total intensities in each ring (in Fourier space) i.e,

$$FRC_{xy}(r_i) = \frac{\sum_{r \in r_i} F_x(r) F_y(r)^*}{\sqrt{\sum_{r \in r_i} F_x^2(r) \sum_{r \in r_i} F_y^2(r)^*}}$$

The signal at a distance r_i from the center of the Fourier transformed images corresponds to the spatial frequency, $f_1 = \frac{N}{x_i}$, where, N is the number of frequency bins, or pixels in the image. The spatial frequency where the FRC falls below a value of 1/7 is defined as cut-off frequency and interpreted as resolution of the full image.

Supplementary 5. Calibration of Exposure Time with Laser Intensity

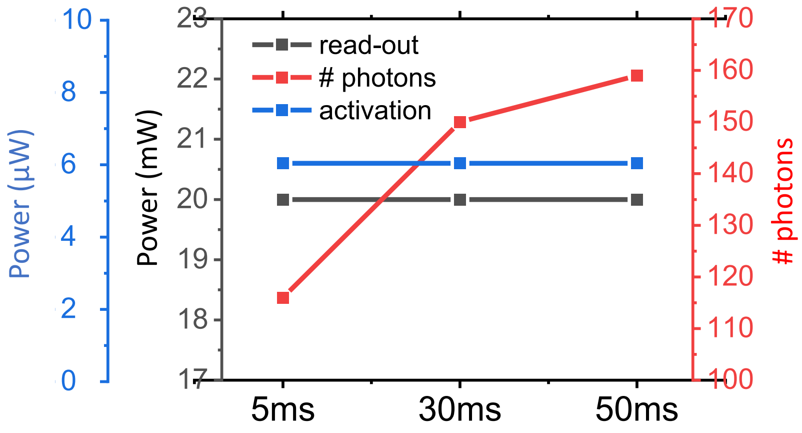

Fig. S5-1. The calibration of exposure time with laser power along with the detected photons.

The calibration of exposure with an activation laser (405 nm) and excitation / read-out laser power is essential for localization microscopy. In Fig. S5-1, we report the details of laser intensities and exposure time used during experimentation. It is evident that the number of emitted photons (mean) reduced substantially (< 120) for 5 ms exposure time, and it gradually reached saturation for increasing exposure time (approximately 160 at 50 ms), even though the intensities were kept constant. The activation and read-out power used during data acquisition was 6 mW and 20 mW, respectively.
